# Engineering peptide-polymer hybrids for targeted repair and protection of cervical lesions

**DOI:** 10.3389/fdmed.2022.1007753

**Published:** 2022-11-25

**Authors:** Paulette Spencer, Qiang Ye, Anil Misra, Josephine R. Chandler, Charles M. Cobb, Candan Tamerler

**Affiliations:** 1 Institute for Bioengineering Research, University of Kansas, Lawrence, KS, United States,; 2 Department of Mechanical Engineering, University of Kansas, Lawrence, KS, United States,; 3 Bioengineering Program, University of Kansas, Lawrence, KS, United States,; 4 Department of Civil Engineering, University of Kansas, Lawrence, KS, United States,; 5 Department of Molecular Biosciences, University of Kansas, Lawrence, KS, United States,; 6 School of Dentistry, Department of Periodontics, University of Missouri-Kansas City, Kansas City, MO, United States

**Keywords:** peptide-mediated remineralization, elderly population, root surfaces, peptide-tethered polymer, cervical, peptide-mediated antimicrobial

## Abstract

By 2060, nearly 100 million people in the U.S. will be over age 65 years. One-third of these older adults will have root caries, and nearly 80% will have dental erosion. These conditions can cause pain and loss of tooth structure that interfere with eating, speaking, sleeping, and quality of life. Current treatments for root caries and dental erosion have produced unreliable results. For example, the glass-ionomer-cement or composite-resin restorations used to treat these lesions have annual failure rates of 44% and 17%, respectively. These limitations and the pressing need to treat these conditions in the aging population are driving a focus on microinvasive strategies, such as sealants and varnishes. Sealants can inhibit caries on coronal surfaces, but they are ineffective for root caries. For healthy, functionally independent elders, chlorhexidine varnish applied every 3 months inhibits root caries, but this bitter-tasting varnish stains the teeth. Fluoride gel inhibits root caries, but requires prescriptions and daily use, which may not be feasible for some older patients. Silver diamine fluoride can both arrest and inhibit root caries but stains the treated tooth surface black. The limitations of current approaches and high prevalence of root caries and dental erosion in the aging population create an urgent need for microinvasive therapies that can: (a) remineralize damaged dentin; (b) inhibit bacterial activity; and (c) provide durable protection for the root surface. Since cavitated and non-cavitated root lesions are difficult to distinguish, optimal approaches will treat both. This review will explore the multi-factorial elements that contribute to root surface lesions and discuss a multi-pronged strategy to both repair and protect root surfaces. The strategy integrates engineered peptides, novel polymer chemistry, multi-scale structure/property characterization and predictive modeling to develop a durable, microinvasive treatment for root surface lesions.

## Contribution to the field statement

By 2060, nearly 100 million people in the U.S. will be over age 65 years. Nearly 30 million of these older adults will have root caries and about 80 million will have dental erosion. These conditions can cause pain and loss of tooth structure that can interfere with eating, speaking, sleeping, and quality of life. Several factors lead to older adults significantly increased risk of root caries and dental erosion. Gingival recession becomes more prevalent with age, increasing tooth-root exposure and the risk of irreversible damage to the tooth. Altered salivary composition or reduced salivary flow can increase caries risk; numerous diseases and medications common in aging populations carry an attendant risk of salivary dysfunction. Elderly patients in settings where regular dental care is not possible are especially susceptible to root caries. Current treatments for root caries and dental erosion have produced unreliable results—these limitations and the pressing need to treat these conditions in the aging population are driving a focus on microinvasive strategies. Drawing on discoveries in peptide engineering, polymer chemistry, and predictive modeling, we propose a multi-pronged strategy to remineralize damaged dentin, inhibit bacterial activity and provide durable protection for root surface lesions.

## Introduction

### Clinical need

Older people have a significantly increased risk of poor oral health and untreated dental disease (1, 2). Treatment costs are often a substantial barrier to dental care for older adults. Reduced mobility and lack of convenient access to care can add to the challenges of accessing dental treatment. Access to appropriate oral health care can be particularly challenging for older adults who are cognitively impaired or living in long-term care facilities (2). A chronic lack of access to oral health care translates to high levels of untreated dental disease for older adults (1, 2). This problem is expected to grow as more working-age adults transition into retirement and potentially, lose their employer-provided dental insurance (2).

By 2060, the Census Bureau projects the U.S. population will grow to 404 million people, a quarter of whom will be over 65 years. One-third of these older adults will have root caries (3) and nearly 80% will have dental erosion (4). These conditions can cause pain, discomfort and loss of tooth structure that can interfere with eating, speaking, sleeping, smiling, socializing and quality of life.

The increased risk of dental erosion and root caries in older adults has been linked to several factors including gingival recession. Gingival recession becomes more prevalent with age, increasing tooth-root exposure and the risk of irreversible damage to the tooth structure (5–7). Tooth-root exposure means that dentin and cementum are exposed to the oral environment. Cementum is sufficiently porous to allow diffusion of acids and enzymes derived from saliva, gingival crevicular fluid and bacteria (8). Dentin is also a porous composite material and loss of mineral as a result of acid and enzyme exposure increases the porosity (9). Over time, acidic foods, beverages, occlusal stress, and abrasion lead to erosion of the exposed tooth-root.

The root caries process starts when acid released by bacteria causes mineral to dissolve (9) and the composition of dentin and cementum increases the risk of mineral dissolution. As opposed to enamel with a composition (by weight) of more than 96% inorganic and 4% organic, the inorganic phase in dentin and cementum is much lower. Dentin is 65% inorganic minerals, 35% organic matrix and water while cementum, the outer mineral tissue layer of tooth-root, is 50%–55% inorganic, 45%–50% organic and water (10). Type I collagen constitutes about 90% of the organic phase for both dentin and cementum (10). If demineralization is not intercepted or stymied by remineralization at the early stages of the caries process, destruction of the inorganic and organic phases will progress leading to cavitation (10).

Reduced salivary flow or altered salivary composition can increase caries risk, and numerous diseases common in aging populations, such as hypertension, diabetes, Alzheimer’s disease, Parkinson’s disease, stroke, and rheumatoid arthritis carry an attendant risk of salivary dysfunction. In addition, a wide variety of medications, including antihypertensives, antidepressants, tranquilizers, diuretics, and antihistamines, also contribute to salivary dysfunction.

### Targeted microorganisms

Microorganisms in the oral cavity exist mainly as biofilms on saliva-coated surfaces such as teeth and restorative materials. Adhesion of primary microorganisms to such surfaces is a key interaction in the initiation of biofilm development. This initial colonization involves interaction of bacterial cell-surface proteins with dental pellicle, a 0.1–1.0 μm thick acellular layer rich in mucinous glycoproteins (11).

*Streptococcus mutans,* a Gram-positive, facultatively anaerobic microorganism, is among the first colonizers of the pellicle. The adhesion of this “pioneer” organism creates an environment that promotes binding of other oral bacteria (12, 13). Ultimately, these activities lead to the formation of a micro-ecosystem (biofilm). *S. mutans* is a primary causative agent of dental caries—higher counts of *Streptococcus mutans* or lactobacilli species are noted with higher prevalence of root caries (7). *S. mutans* is both a “pioneer” organism in biofilm formation and produces acids and enzymes that damage the tooth.

In addition to the high prevalence of root caries and dental erosion, nearly 60% of the population 65 years and older will have periodontal disease (14). *Porphyromonas gingivalis,* a Gram-negative anaerobic microorganism, plays a prominent role in inflammatory periodontal disease such as periodontitis (15). Periodontitis is characterized by destruction of the periodontal ligament, and alveolar bone. *P. gingivalis* can attach to a variety of substrates in the oral cavity including synthetic materials, soft tissues and other bacteria (16). Furthermore, *P. gingivalis* expresses virulence factors that allow it to evade host responses and to colonize and spread within the tissues (17). It is theorized that periodontal inflammation results from the interaction of the host immune system and a dysbiotic subgingival biofilm (8). Dysbiosis potentially results from the interaction of “keystone” organisms such as *Porphyromonas gingivalis* and *Filifactor alocis* with subgingival pathogenic microbes (8).

### Quorum sensing

Many bacteria communicate with one another using chemical signaling molecules that may be beneficial in mixed microbial communities (18). A particularly well understood type of cell-cell communication is quorum sensing. Quorum-sensing systems become activated at a critical density or “quorum” and cause changes in gene expression that switch on certain group activities. For example, *P. gingivalis* uses the AI-2 quorum-sensing signal to switch on biofilm formation. AI-2 signals are synthesized by the LuxS signal synthase, which cleaves S-ribosylhomocysteine into 4,5-dihydroxy-2,3-pentanedione (DPD), which further derivatizes to become AI-2 (19). Many different bacteria have LuxS enzymes and can produce AI-2, thus AI-2 may be important for coordinating interactions between different species. In *P. gingivalis*, AI-2 is necessary for the formation of mixed biofilms with other species such as *Streptococcus gordonii* (20). *S. mutans* also encodes a *luxS* AI-2 synthase gene that is important for biofilm formation (21). Thus AI-2 might be an essential cell-cell communication molecule that is used for coordinating formation of mixed biofilms in the oral cavity.

In addition to AI-2, *S. mutans* encodes a second quorum sensing system that relies on a signal called competence stimulating peptide (CSF) (22). Unlike AI-2, which is found in many species, CSF is found only in *S. mutans*. CSF is a post-translationally modified 21 amino acid peptide that is encoded by the *comC* gene. Other genes involved in CSF signaling are *comAB*, which code for the CSF secretion apparatus, and *comDE*, which code for the membrane-localized CSF receptor and cognate response regulator, respectively. At a sufficient population density CSF signaling induces natural competence, which is the uptake of DNA from the environment. Natural competence may be important for acquiring DNA that has been released from nearby bacteria, such as antibiotic resistance genes that might be beneficial in certain conditions. CSF has also been shown to regulate other behaviors, such as production of antimicrobials. Thus, CSF is another type of quorum-sensing system that may be important for competing with other strains or species in polymicrobial communities.

Quorum sensing has been the target of much effort to develop novel therapeutics that function by blocking critical cell-cell interactions rather than essential functions that are targeted by classic antibiotic approaches. These types of “anti-virulence” therapeutics are novel in concept because they may block destructive properties of the microbes (e.g., establishment of caries infections) but do not block essential processes that are needed to survive. It is thought that such anti-virulence therapeutics might escape the normal selective pressures that quickly lead to resistance in the case of classic antibiotic therapies, and ultimately be a more effective treatment strategy. However, basic studies of such therapeutics are lacking, particularly studies in the context of more complex multi-species and multi-strain communities, and such studies are needed to demonstrate their efficacy and determine the best strategies for treating infections.

### Non-cavitated and cavitated lesions on root surfaces

Cavitated and non-cavitated root carious lesions are difficult to distinguish in practice (23). In general, non-cavitated carious lesions can be described as surfaces that appear macroscopically intact. A cavitated carious lesion presents as a surface with a distinct discontinuity or break in the surface integrity (23).

**FIGURE 1:**
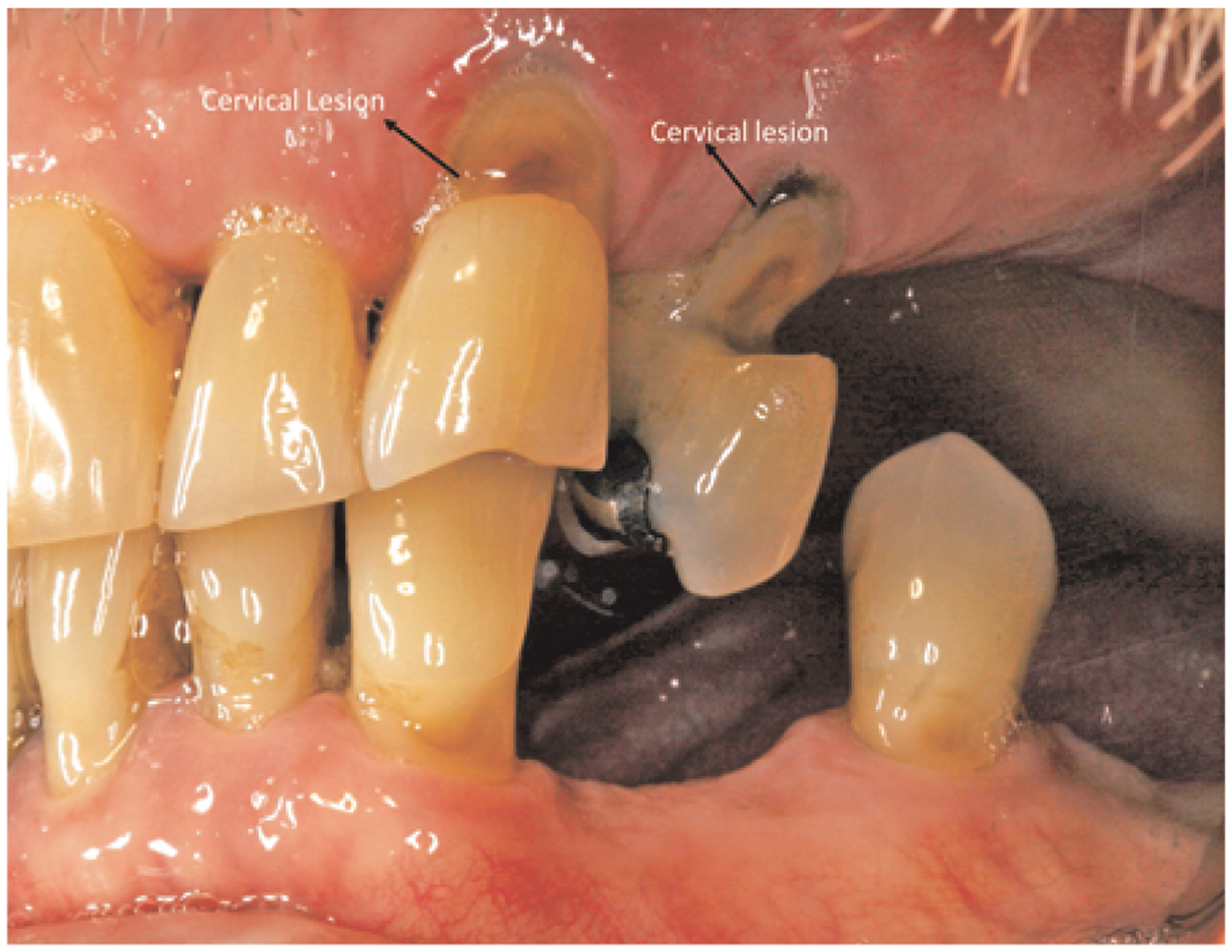
Non-carious cervical lesions.

Non-carious root lesions may be caused by erosion, abrasion and/or occlusal stress (24). (Figure 1: Clinical Image) Restoration of non-carious root lesions may be required to relieve hypersensitivity, to prevent further loss of tooth structure, and to improve esthetics (24, 25).

**FIGURE 2:**
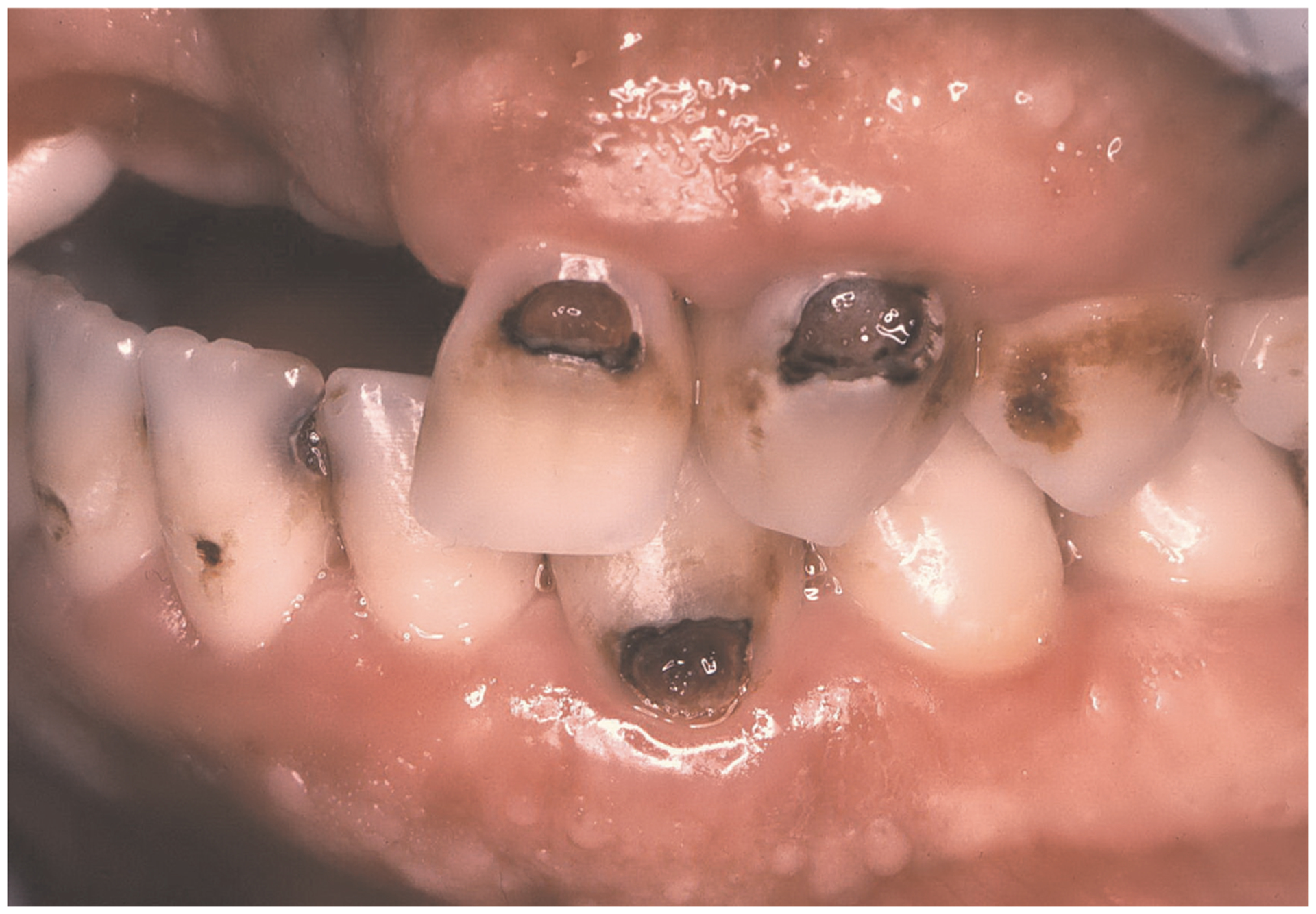
Clinical image of root caries.

Root caries is a cavitation occurring below the cementoenamel junction that involves both cementum and dentin but not enamel (7). (Figure 2: Clinical Image) These lesions are insidious—overhanging enamel leads to surfaces that are not readily accessible for cleaning. The overhanging enamel provides a niche that collects and retains food particles, e.g., fermentable carbohydrates—bacteria will thrive in these nutrient-rich environments. Dentin and cementum will be exposed to low-pH biofilm for extended periods (26, 27). As noted recently, the following factors are associated with an increased risk of root cares: older people, lower socioeconomic status, gingival recession, tooth root exposure, tobacco use and poor oral hygiene (7). Elderly patients with compromised functional capabilities or in settings where regular dental care is not possible are especially susceptible to root caries.

## Current treatments

Root-surface lesions are more challenging to treat than those at the coronal surface. The efficacy of glass-ionomer-cement or composite-resin restorations is hampered by moisture-control difficulties and the non-retentive saucer-shaped cavity preparations that are often required to conserve tooth-root structure (26, 27). The restoration will generally be bonded to dentin and/or cementum—dentin and cementum are particularly challenging substrates for bonding (24). For example, in non-carious cervical lesions a large fraction of the substrate may be sclerotic dentin—sclerotic dentin is resistant to acid-etching which is commonly used with adhesive bonding. Inadequate bonding leads to a fragile adhesive seal—a seal that is readily damaged by acids, enzymes, and oral fluids.

Inadequate bonding, non-retentive saucer-shaped cavity preparations and moisture contamination contribute to the high failure rates of root-surface glass-ionomer-cement or composite-resin restorations—annual failure rates are 44% and 17%, respectively (26). The repair of cervical lesions with proximal extensions is exceptionally challenging—the repair of these lesions requires extensive removal of sound tooth structure (25). The longevity of these restorations is significantly shortened (25). The failures lead to a vicious cycle of repeated restorations, with each cycle weakening the tooth and increasing the risk of endodontic treatment or extraction.

Given the prevalence of root-surface lesions in the aging population, these limitations are driving a focus on microinvasive strategies to arrest or reverse the carious lesions. Fluoride gel (5,000 parts-per-million) inhibits root caries, but success requires filling prescriptions and daily use, which may not be feasible for some older patients (23, 28). For healthy, functionally independent elders, chlorhexidine varnish applied every 3 months inhibits root caries, but this varnish stains the teeth brown and its bitter taste discourages regimen compliance (28). Silver diamine fluoride (SDF) can arrest and inhibit root caries, but stains the teeth black (23, 28).

## Next generation treatments: peptide-polymer hybrids

There is increasing interest in approaches that integrate biologically instructive roles into dental biomaterials—the ultimate goal of these approaches is to prevent oral disease and/or restore oral health (29–31). Biomolecules are essential in physiological processes, and smaller molecules such as peptides offer the potential to mimic their function while also, serving as uniquely versatile molecular tools to couple biologically instructive roles in material systems (32, 33). Our group as well as others have explored peptide-based approaches to repair mineralized dental tissues damaged by caries, trauma or periodontal diseases and derivatized dental materials with a wide range of bioactivities including antimicrobial activity to prevent infection (29, 34–38).

Our initial efforts focused on mineralization as next generation treatment options for dental biomaterials. Dental enamel is the hardest and most highly mineralized tissue—enamel can withstand 770 N and nearly one million cycles per year (29). Enamel biomineralization has been an inspiration for scientists to understand the amelogenesis, i.e., enamel formation process and to replicate this intricate biological process. Amelogenin protein, a major constituent of the developing enamel matrix, plays a critical role in the oriented growth of enamel mineral (39–41). There have been several studies to correlate the functional domains of amelogenin and utilize this knowledge to remineralize and restore tooth structure using biomolecules as a biomimetic guide. To develop amelogenin derived functional peptides, we developed a bioinformatics scoring matrix approach to identify short amino acid similarity regions across the full length amelogenin protein. In this search, we used our combinatorial phage display library of selected calcium phosphate binding peptides (42). In our prior research, we demonstrated that these peptides can mediate remineralization function and remineralize a cementum-like hydroxyapatite mineral layer on demineralized root dentin (34, 43).

Another active area of peptide incorporation in the dental biomaterials focuses on antimicrobial peptides (AMPs) to prevent infection. With the rapidly proliferating concern on antibiotic resistant strains, the non-antibiotic-based approaches are an active area of research. AMPs gained significant attention as small molecules that are an integral part of host defense systems found among all life forms. These small peptides have excellent antibacterial, antibiofilm properties, and low bacterial strain resistance. We engineered biomaterials surfaces with AMPs and developed localized delivery approaches for their easy deployments on biomaterials including dental implants (44, 45). We developed machine learning approaches to classify antimicrobial peptides and enrich sequence domains for effective antimicrobial search with enhanced properties (46, 47).

Resin-based composite restorations are among the most commonly applied restorative materials used to treat defective tooth tissues. With their improved formulations, composites have become increasingly popular in dentistry, however they suffer from recurrent dental caries occurring at the margin between the composite and the tooth. The cariogenic bacteria, S. mutans, adheres to the tooth/adhesive/composite interface and creates a microenvironment promoting the subsequent attachment and growth of bacteria leading to biofilm formation. Acidic environment produced by the bacterial activity demineralizes the tooth surface and erodes the dental adhesive resulting in enlargements of the gaps between the tooth and the composite. Despite dental adhesive polymers possessing broad and versatile properties, they lack biological functionalities such as remineralization and antimicrobial. Building on our prior research (46–55), we developed a multi-pronged strategy to both repair and protect exposed dentin (50). This multi-pronged strategy: (a) remineralizes damaged dentin; (b) inhibits bacterial attack; and (c) provides durable protection. Since cavitated and non-cavitated root lesions are difficult to distinguish, our multi-pronged, microinvasive strategy will be suitable for treating both lesion types.

**FIGURE 3:**
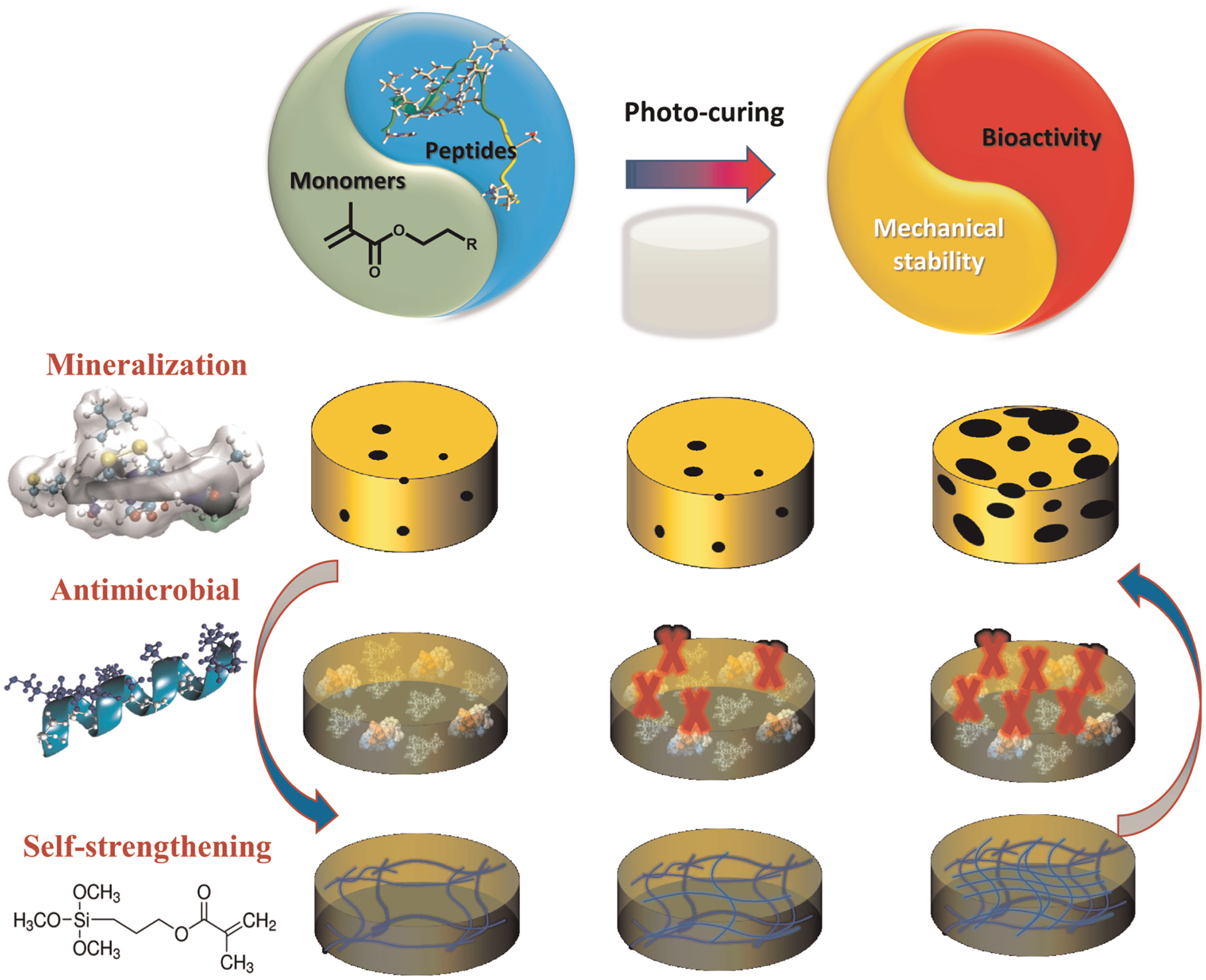
Schematic of peptide-polymer hybrid system.

**FIGURE 4:**
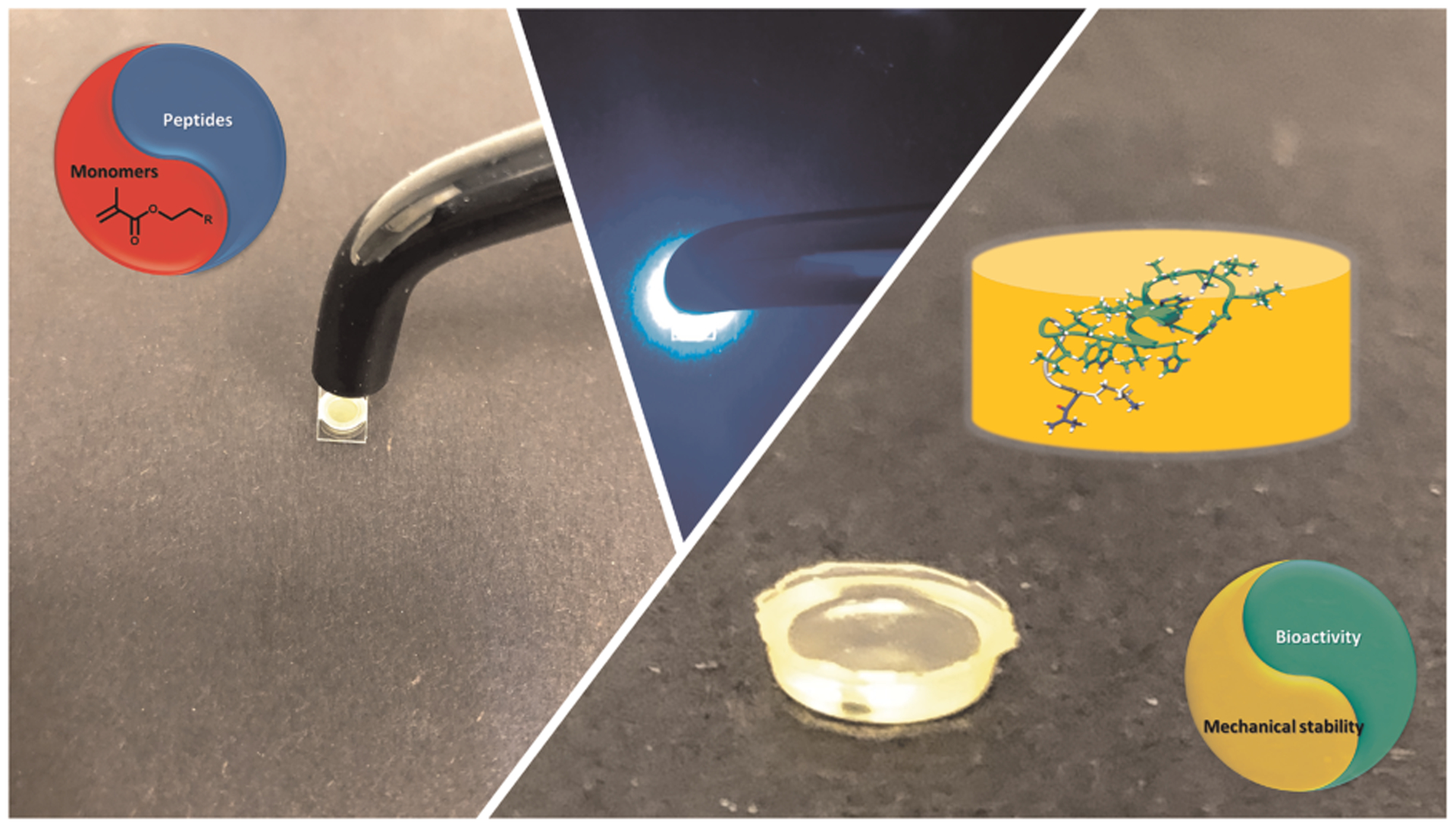
Formulation polymerized with dental curing light to produce polymer with tethered peptides to provide bioactive cues to inhibit bacterial activity and promote remineralization.

Polymer-peptide conjugates are generally hybrid soft materials, which are designed to achieve synergistic behavior of both components. There have been several studies on hydrogel-based materials, however low mechanical properties and rapid erosion of these conjugates inhibit their application as dental restorative materials. Our recent investigations have led to a synergistic approach to design a peptide-polymer hybrid system. Our strategy includes polymer-tethered peptides to promote remineralization, polymer-tethered antimicrobial peptides to inhibit S mutans activity, and novel self-strengthening polymers (Figure 3). The alkoxysilane-containing polymers achieve self-strengthening, i.e., intrinsic network reinforcement in both neutral and acidic conditions (56, 57). The intrinsic network reinforcement leads to enhanced mechanical properties and hydrolytic stability (56–59). The tethered peptides present bioactive cues to inhibit bacterial activity and promote remineralization at the lesion site (50) (Figure 4).

### Peptide-mediated remineralization

**FIGURE 5:**
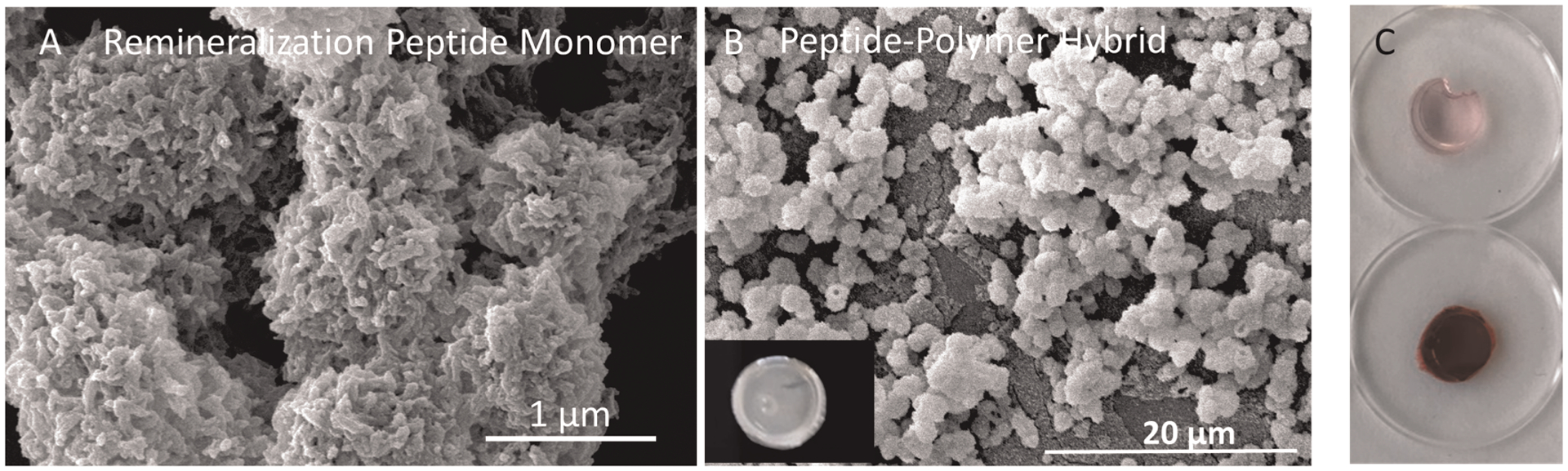
(**A**) SEM images of the minerals formed in the presence of K-GSGGG-HABP monomer; (**B**) K-HABP:AMPM7 integrated polymer disc samples was monitored under SEM after completing the overnight mineralization reaction; (**C**) peptide integrated polymer discs after mineralization were stained with alizarin red. The same type of discs without mineralization were used as controls.

Our prior art built upon incorporating a phage-display-selected hydroxyapatite binding peptide (HABP: CMLPHHGAC) (42). Among the several HABPs identified using the combinatorial library selection, this peptide was shown to bind and simultaneously exhibit control over calcium phosphate mineralization. We demonstrated that this peptide also binds to dentin and mediates the remineralization at the dentin-adhesive interface (53). We also explored our amelogenin derived peptides to provide treatment at the lesion site and demonstrated that peptide-mediated remineralization diffuses into the substrates and can be tuned for increased nucleation sites (55). The repertoire of calcium-phosphate-binding peptides that we developed allows us to develop peptide-polymer hybrids that can be tuned to promote remineralization (Figure 5) of the damaged dentin with different kinetics and morphology as well as availability at the lesion sites (50).

### Antibacterial activity

Agents such as chlorhexidine (CHX), fluoride, and quaternary ammonium salts (QAMs) have been incorporated in dental materials to prevent caries (60–63). The antimicrobial activity of these agents is generally achieved through gradual release—gradual release can lead to inconsistent dosage and short-term effectiveness. In addition, these agents can discolor the tooth, interfere with taste (64–66), drive development of bacterial resistance (67, 68), and display toxicity to host tissues (60, 62, 64). Our approach of incorporating antimicrobial peptides (AMPs) offers a therapeutic alternative that can be delivered at the site as an integral part of the polymeric network (50–52, 69, 70). AMPs provide early-stage protection and also bridge between innate and acquired immunity (71). Despite these advances, the vast potential of AMPs is quite limited — the limitation is related, in part, to the concerns associated with their systemic delivery which requires high concentrations and raises toxicity concerns. Our approach provides an alternative delivery strategy to deploy peptides on the sites and increases their availability and preserves their antibacterial efficacy.

### Dual peptide-polymer hybrid

We have developed a dual peptide-polymer hybrid with tunable properties (50). The peptide-polymer hybrid was produced by mixing two peptides, i.e., an antimicrobial peptide and mineralization mediator peptide, tethering them to monomer and co-polymerizing the peptide-tethered monomers. In brief, HABP and AMP were synthesized using oligomeric spacers to tether them to a methacrylic acid (MA) as peptide-monomers. The sequences for the peptide-monomers are: MA-K-GSGGG-CMLPHHGAC and MA-K-GGG-KWKRWWWWR-NH2 for HABP and AMPM7, respectively (50). The peptides become an integral part of the polymer and their antimicrobial and remineralizing properties are displayed simultaneously. The tethered peptides remineralize the damaged tooth structure and inhibit bacterial activity, i.e., S. mutans, while the polymer provides durable protection to the prepared tooth surface.

## Integrated experimental & computational approaches for rational treatment design

The durability of treatments for root surface lesions, and in general, other treatments for dental disease and/or trauma are impacted by a complex bio-chemo-mechanical environment. This complexity is manifold at the interface between the treatments and the native tooth structure.

### Spatial scales

The treatments are characterized by a complex system of different material components—the material components have varying mechanical properties and are arranged in a variety of morphologies. The mechano-morphological properties of these material components can be considered at different spatial and temporal scales. From the viewpoint of spatial scales, material systems can be broadly considered at:

1. The macro-scale defined at the millimeter level spanning 0.1 mm–10 mm. At this scale, the interfaces between native materials and the treatment appears as a thin layer with variable mechano-morphology.
2. The micro-scale defined at the micrometer level spanning 0.1 μm–100 μm. At this scale, the interfaces between native materials and the treatment appears as a complex mechano-morphology construct. Such a micro-mechano-morphology is characterized by ill-defined structure-property relationships, including heterogeneity and/or phase separations, inter-digitation, defects, presence of pore fluid, and others.
3. The atomic-scales defined at the nanometer level spanning 0.1 nm–100 nm. The mechano-morphology at this scale can be surmised to consist of a variety of covalent, ionic and hydrogen bonds among the multi-atomic components, i.e., the native apatitic mineralite, collagen and the introduced treatment materials.

### Temporal scales

From the viewpoint of temporal scales, the complex mechano-morphological composition and the dynamic external loading produced at the occlusal surfaces indicates that the behavior at the interface between the treatment and native structure is both rate- and time-dependent. Thus, the durability and long-term performance of the treated root surface lesions depends upon the rate-dependence (visco-elastic-damage-plastic) behavior as well as the time-related bio-chemo-mechanical transformations of the various material components.

Clearly, the characterization and modeling of treatment constructs pose significant challenges. These challenges are not only for the laboratory analytical techniques, but also for mathematical modeling necessary for informing the design of effective treatments. A practical way to understand the impact of the complex mechano-morphologies at the different spatial and temporal scales is to develop integrated experimental and modeling approaches. For example, we have discussed the spatial scales issues for dentin-adhesive (d-a) interfaces using macro- and micro-scale elastic models and the temporal issues using micro-scale visco-elastic-damage-plastic models [see discussion in (72)]. Similarly, experimental techniques at appropriate scales may be used to characterize mechano-morphology properties of the native materials as discussed in the following references (73–77).

Indeed, appropriate mathematical models can be a significant component of the iterative scheme for developing effective treatments. Along this line, we have been developing mathematical models of bio-chemo-mechanical behavior of adhesives and their performances at the d-a interface. For tractable analyses of this highly complex multi-scalar construct, we have developed methods applicable at different spatial-scales, time- and rate- dependency. We have applied this approach to connect molecular-level data to model time- and rate-dependent stress/strain relationships in adhesives, as well as in adhesive-collagen constructs such as hybrid-layer mimics (58, 59, 73–75, 78, 79).

The models exploit recent developments in continuum physics that aim to connect mechanisms at nano- and micro-scales to larger scales (80–83). These models aid the rational design of our adhesive formulation by predicting their constitutive behaviors under relevant oral conditions (73–75, 78, 79, 84–86). Further, using μ-scale structure-property measurements (87) we have developed 3D Finite Element (μFE) models (87–90). The μFE models can be used to assess how the change in adhesive properties effects the performance of the d-a interface by directly utilizing the predicted constitutive behaviors.

The stress analysis at macro-scales (tooth scale) can only assess occlusal-loading stresses at the d-a interface, while micro-scale stress analysis identifies the locations where stresses concentrate. The (μFE) models have been used to perform micromechanical stress analyses (88–90). These analyses show that the different material phases at the d-a interface experience different stress amplitudes under functional load (88, 89). As a result, the different material phases reach their failure strengths at different overall stress levels. Thus, the overall failure behavior of the d-a interface is not necessarily determined by the weakest component—instead, failure is determined by the component whose stress concentration is closest to its failure strength (90).

**FIGURE 6:**
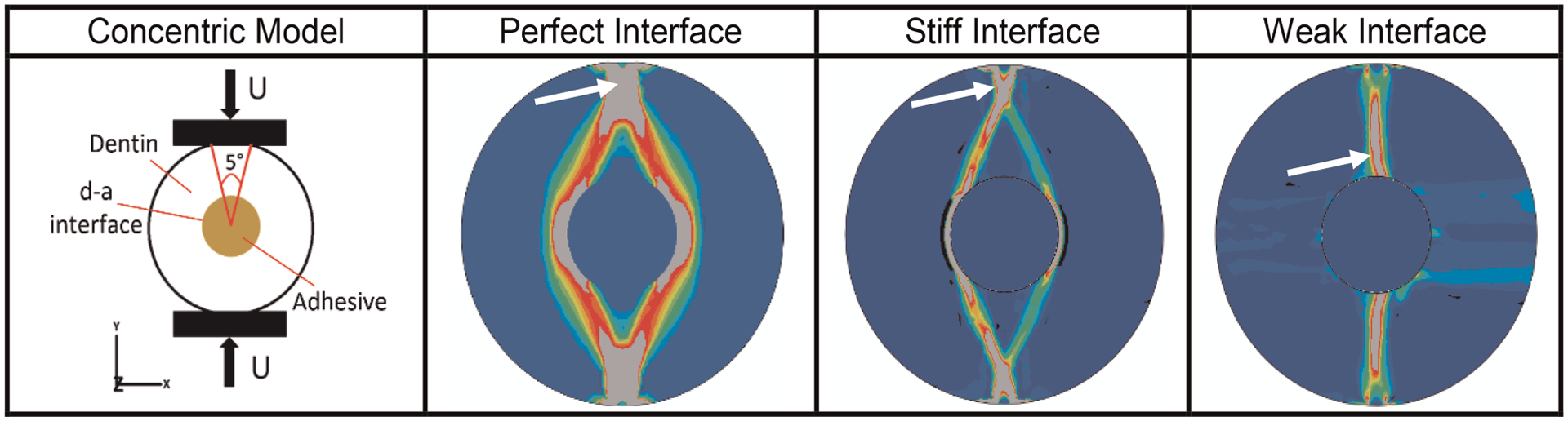
Results from μmechanical stress-analysiaas of d-a interface for different type of potential interfacial conditions based upon finite element in-silico analyses of a concentric model of d-a interface formed by creating a hole in a cylindrical dentin sample. Panel 1 shows the schematic of the concentric model and its external loading regime. Panels 2 to 4 give the octahedral shear strain (blue-low, red-high) at post failure showing fracture zone (gray – indicated by white arrow) for three ideal cases of d-a interfacial conditions. For perfect and stiff interfaces, respectively, the fracture initiates from the load application zone on the sample periphery and propagates towards the interface, while for a weak interface, the fracture initiates from inside at the d-a interface and propagates outwards towards the loading location.

We have also used μmechanical stress-analysis (Figure 6) to show the effects of such stress concentrations on the mechanisms that govern overall fatigue failure behavior of the d-a interface (82). Predictions were compared to experimental data to illustrate the predictive power of our methodology (91). The modeling is used to predict properties and forecast behavior of the adhesive under conditions relevant to function in the mouth. Feedback from the modeling informs refinement of the polymer formulations and promotes targeted design optimization of the adhesive.

Feasible integrated, iterative experimental and mathematical modelling approaches are required to develop a comprehensive understanding of how treatments perform in function. For example, to address the issues related to spatial scales by using independent macro- and micro-scale models as well as temporal issues by developing rate-dependent models—these models as well as experimental validation are necessary for a comprehensive understanding. The current efforts, which are typically based upon classical elastic continuum mechanics or experimental approaches based upon the classical theories are inadequate. Refined modeling and experiments, that aim to bridge the response at various scales such as the micromechanically based models that have the capability of connecting the micro-scale mechanisms to the macro-scale phenomena (82, 85, 92, 93) can advance our understanding of the effectiveness of proposed treatments.

## Summary

By 2060, 100 million Americans will be over 65 and one-third of these older adults will have root caries and nearly 80% will have dental erosion—conditions that cause pain and loss of tooth structure that can interfere with eating, speaking, sleeping and quality of life. Several factors lead to older adults’ significantly increased risk of root caries and dental erosion. Gingival recession becomes more prevalent with age, increasing tooth-root exposure and the risk of irreversible damage to the tooth. Reduced salivary flow or altered salivary composition can increase caries risk, and numerous diseases as well as medications common in aging populations contribute to salivary dysfunction.

Current restorative treatments for root caries and dental erosion have produced unreliable results such as high annual failure rates. These limitations and the pressing need to treat these conditions in the aging population are driving a focus on microinvasive strategies, such as sealants, varnishes, and gels. Sealants are effective for coronal caries, but ineffective for root caries. The frequent application required for chlorhexidine varnish and fluoride gels may not be feasible for elders with compromised functional capabilities or individuals living in settings where regular dental care is not possible. The bitter-tasting chlorhexidine varnish must be applied every 3 months. Fluoride gels require prescriptions and daily use. Silver diamine fluoride can both arrest and inhibit root caries but stains the treated tooth surface black.

The limitations of current approaches as well as the high prevalence of root caries and dental erosion in the aging population create an urgent need for alternative treatments. Peptide-polymer hybrids offer a multi-pronged, microinvasive strategy for repairing and protecting exposed tooth-root surfaces. This multi-pronged strategy includes: (a) polymer-tethered peptides to promote remineralization of damaged dentin; (b) polymer-tethered antimicrobial peptides to inhibit *S mutans*; and (c) self-strengthening polymers to provide durable protection. The tethered peptides present bioactive cues to promote remineralization and inhibit bacterial activity at the lesion site without staining the treated surface black. The polymers offer hydrolytic stability and enhanced mechanical properties under both neutral and acidic conditions.

While the peptide-polymer hybrids offer promise, the design and production of these materials pose significant challenges. For example, the properties of polymers depend on a large parameter space and optimization of these properties is typically a laborious, iterative process. Features such as composition, polymerization, and processing parameters are systematically changed and the effects determined by measuring the properties of the new polymer. This process will only become more intractable as additional biological parameters are included. One approach to these challenges is multi-scale characterization coupled with modeling to provide insights beyond what could be accomplished if either of the approaches were applied independently. With their ability to enable *in silico* parameter optimization, computational models to correlate system parameters with material properties can be indispensable for streamlining this process.

Finally, there are multiple unresolved questions and challenges that must be addressed to meet the unprecedented demand for new restorative treatments for root caries and dental erosion. These questions and challenges include a reliable, reproducible, and quantitative understanding of the substrate at atomic, molecular and macro-scales. Diagnostic tools that can assess the extent of damaged dentin *in vivo*. Laboratory results must be validated by representative and reproducible *in vivo* models (94). In vitro models that predict degradation must be calibrated with clinical data (94). Advanced imaging modalities that can reveal subtle compositional differences that occur in the substrate, i.e., dentin and cementum, and restorative material during clinical function are required (94, 95). Multiscale imaging, advanced technologies, and computational modeling are required to measure the interactions of materials with the tissue microenvironment (96).
